# Chest X-Ray Features in 130 Patients with Bronchiectasis

**DOI:** 10.3390/diseases12120323

**Published:** 2024-12-10

**Authors:** Hikaru Sawada, Ryohei Kudoh, Atsushi Yokoyama, Akihiko Hagiwara, Kazufumi Hiramatsu, Jun-ichi Kadota, Kosaku Komiya

**Affiliations:** 1 Respiratory Medicine and Infectious Diseases, Oita University Faculty of Medicine, 1-1 Idaigaoka, Hasama-machi, Yufu, Oita 879-5593, Japan; 2 Department of Respiratory Medicine, Oita Prefectural Hospital, 2-8-1 Bunyo, Oita 870-8511, Japan; 3 Research Center for GLOBAL and LOCAL Infectious Diseases, Oita University Faculty of Medicine, 1-1 Idaigaoka, Hasama-machi, Yufu, Oita 879-5593, Japan

**Keywords:** bronchiectasis, postinfection, nontuberculous mycobacterium, pneumonia

## Abstract

*Background/Objectives:* The prevalence of bronchiectasis is increasing globally, and early detection using chest imaging has been encouraged to improve its prognosis. However, the sensitivity of a chest X-ray as a screening tool remains unclear. This study examined the chest X-ray features predictive of bronchiectasis. *Methods:* We retrospectively reviewed the chest X-rays of patients with bronchiectasis diagnosed using high-resolution computed tomography who visited our institute from January 2013 to March 2020. Patients with cardiac pacemakers, lung cancer, and interstitial pneumonia, which might bias the detection of bronchiectasis, were excluded. Two respiratory physicians independently determined the presence or absence of potential features reflecting bronchiectasis, including a vague cardiac silhouette on chest X-rays. *Results:* The study enrolled 130 patients, including 72 women (55.4%), with a mean age of 72 years. The features observed on chest X-rays included granular shadows (88.5%, n = 115), vague cardiac silhouettes (48.5%, n = 64), nodular shadows (45.4%, n = 59), a tram-track appearance (35.4%, n = 46), pleural thickening (26.9%, n = 35), vague diaphragm silhouettes (25.4%, n = 33), and a ring sign (24.6%, n = 32). The kappa values for these features were 0.271, 0.344, 0.646, 0.256, 0.312, 0.514, and 0.376, respectively. *Conclusions:* Although traditional chest X-ray features believed to reflect bronchiectasis, such as the tram-track appearance or ring sign, were not frequently seen, vague cardiac silhouettes and granular shadows had high positivity rates, indicating their potential utility for bronchiectasis screening. However, the interobserver concordance rates were unsatisfactory.

## 1. Introduction

Bronchiectasis is a chronic respiratory disease characterized by irreversible abnormal dilation of bronchi and thickening of bronchial walls [1]. Patients with bronchiectasis typically complain of a persistent cough and sputum production. In a retrospective analysis of 14 million people using the Clinical Practice Research Datalink in the UK [2], the prevalence rate (per 100,000 persons) increased from 350.5 in 2004 to 566.1 in 2013 for females and from 301.2 in 2004 to 485.5 in 2013 for males. According to the report on the epidemiology of middle lobe syndrome, a representative type of bronchiectasis, among patients undergoing medical checkups in Shizuoka prefecture, Japan, from 1992 to 1993 [3], the prevalence was estimated to be approximately 170 cases per 100,000 persons.

The underlying causes of bronchiectasis vary widely and differ by country or region. The European bronchiectasis registry including 16,963 cases found that bronchiectasis was most commonly idiopathic (38.1%), with other causes including infection (21.2%), chronic obstructive pulmonary disease (COPD; 8.1%), and asthma (6.9%) [4]. According to a registry study of bronchiectasis covering 13 US clinical facilities [5], although the data permitted overlap, the causes were postinfection (68%), nontuberculous mycobacterial (NTM) disease (67%), gastroesophageal reflux disease (47%), asthma (29%), and COPD (20%). NTM lung disease is considered the major cause of bronchiectasis in Japan, accounting for 78.5% of cases based on a report by Asakura et al., who prospectively collected data on patients with bronchiectasis attributable to NTM disease or non-cystic fibrosis from 12 hospitals [6].

Bronchiectasis is diagnosed according to the presence of at least one of the following findings on chest high-resolution computed tomography (HRCT): bronchoarterial ratio > 1.0, lack of tapering, and airway visibility within 1 cm of the costal pleural surface or touching the mediastinal pleura [7]. Therefore, HRCT-based assessments are essential for diagnosing bronchiectasis [8], but HRCT cannot be feasibly performed for all patients with complaints of respiratory symptoms. Bronchiectasis can lead to recurrent infection, and it is a significantly poor prognostic factor among patients with cystic fibrosis [9]. Hence, early diagnosis of bronchiectasis through screening tests is required to treat the underlying diseases. Chest X-rays are expected to be a useful screening tool in the early diagnosis of bronchiectasis, and several features are believed to have high specificity in diagnosing this disease [10]. These features include the “tram-track appearance”, in which the bronchial wall appears as two parallel lines because of the circumferential thickening and lack of tapering, and the “ring sign”, reflecting the cross-section of the bronchus with a thickened wall. In addition, in cases of bronchiectasis associated with infection, sputum might accumulate in the dilated bronchi, resulting in features such as the “finger-in-glove sign” and “air–fluid level”. However, these features can be present in other lung diseases such as allergic bronchopulmonary mycosis or lung abscess [11,12], conferring them low specificity for detecting bronchiectasis.

When considering the utility of screening tools, the sensitivity of each finding should be evaluated. Currie et al. reported that in patients diagnosed with bronchiectasis by bronchography, the positive rate for each feature on a chest X-ray was approximately 20%, and the proportion of patients with at least one feature was 47% [13]. As previously mentioned, NTM lung disease is one of the major etiologies of bronchiectasis. Because the bronchiectasis caused by this disease predominantly distributes in the middle lobe and lingular segment, a vague cardiac silhouette and diaphragm can be frequently observed on chest X-rays. Furthermore, NTM forms multiple granulomas in the lungs, which might present as granular shadows on chest X-rays. However, no study focusing on the usefulness of these features in detecting bronchiectasis has been conducted. If these findings display high sensitivity, then a chest X-ray would be a useful tool for bronchiectasis screening. Therefore, this study examined the prevalence of abnormal features in chest X-rays, including the vague cardiac silhouette and diaphragm, among patients with bronchiectasis diagnosed using HRCT.

## 2. Materials and Methods

### 2.1. Study Design

This was a retrospective, cross-sectional study. Of the 172 patients who visited Oita University Hospital from January 2013 to March 2020, received a diagnosis of bronchiectasis using HRCT, and underwent bronchoscopy to evaluate the causative underlying diseases, 130 patients were included in this study after excluding those with lung cancer, interstitial pneumonia, and implanted devices such as cardiac pacemakers, which can interfere with the interpretation of X-rays. If multiple chest X-rays were taken, the most recent image taken within 3 months prior to bronchoscopy was analyzed.

### 2.2. Definitions and Radiological Evaluation

Bronchiectasis was defined as a bronchoarterial ratio > 1.0 on HRCT, and the lobe in which the features were observed was recorded. Its location was classified into six lobes with the lingular segment of the left upper lobe considered a separate lobe. The field on chest X-rays was divided into three levels on the left and right sides: cranial to the second rib, caudal to the fourth rib, and the intermediate area. From each area, the presence or absence of the following 11 features was determined: tram-track appearance [10,14], ring sign [10,14], air–fluid level [14], finger-in-glove sign [10], vague diaphragm, vague cardiac silhouette, pleural thickening, atelectasis, granular shadows, nodular shadows, and lung volume loss. The chest X-rays were independently evaluated by two respiratory medicine residents, and the kappa values for each interpretation were calculated. If there was disagreement between the features in the two evaluations, a third interpretation was requested from a respiratory medicine specialist to confirm the features. If the location of the features did not match, then the location was determined through discussion. Furthermore, this study collected patients’ demographic information, including age, sex, body mass index, FEV1% predicted, and colonization by *Pseudomonas aeruginosa*. We attempted to calculate the FACED score as a sensitivity index for bronchiectasis [15]; however, the mMRC scale was not routinely documented in our institution. Thus, the other parameters used to calculate the FACED score were extracted.

### 2.3. Ethical Approval

This study was approved by the Institutional Ethics Committee of Oita University, Faculty of Medicine (approval no. 2739; approval date: 27 February 2024). All aspects of this study complied with the principles of the Declaration of Helsinki. The requirement for informed consent was waived by the committee owing to the retrospective design of the study. The information for this study was posted at the hospital.

## 3. Results

The 130 enrolled patients included 72 women (55.4%), and the median patient age was 72 years (interquartile range = 64–77). The median body mass index and FEV1% predicted, which are included in the FACED score, were 19.2 (interquartile range = 17.3–21.5) and 92.5 (interquartile range = 76.3–103.1), respectively. The most common causes of bronchiectasis were idiopathic, followed by NTM lung disease, pulmonary aspergillosis, rheumatoid arthritis, tuberculosis, and nocardiosis in 70 (53.8%), 37 (28.5%), 8 (6.2%), 4 (3.1%), and 2 patients (1.5%), respectively. Diffuse panbronchiolitis, sclerosing neurocytoma, pulmonary malt lymphoma, paragonimiasis, and *Pseudomonas aeruginosa* infection were the causes in one patient each (0.8%). Consistent with previous reports [6,16], most cases occurred after infection, including those associated with NTM lung disease or idiopathic causes.

**Figure 1:**
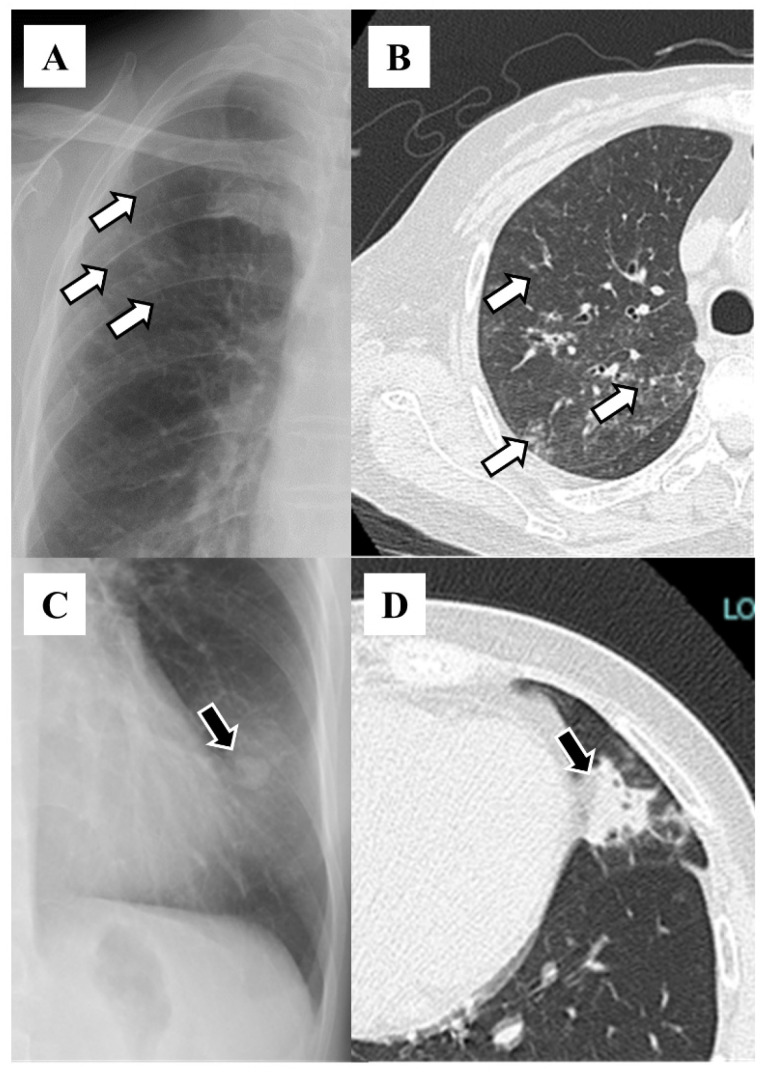
Granular shadow on chest X-ray (**A**) and high-resolution computed tomography (**B**). White arrows indicate the same lesions within the patient. Nodular shadow on chest X-ray (**C**) and high-resolution computed tomography (**D**). Black arrows indicate the same lesions within the patient.

**Figure 2:**
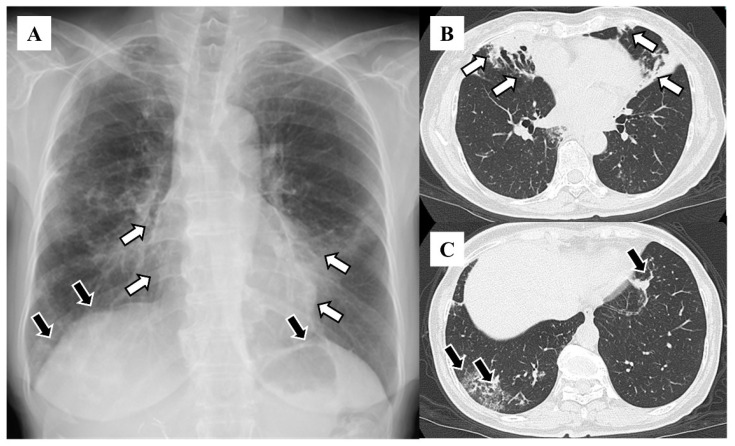
Vague cardiac silhouette on chest X-ray (**A**) and high-resolution computed tomography (**B**). White arrows indicate the same lesions within the patient. Vague diaphragm on chest X-ray (**A**) and high-resolution computed tomography (**C**). Black arrows indicate the same lesions within the patient.

**Figure 3:**
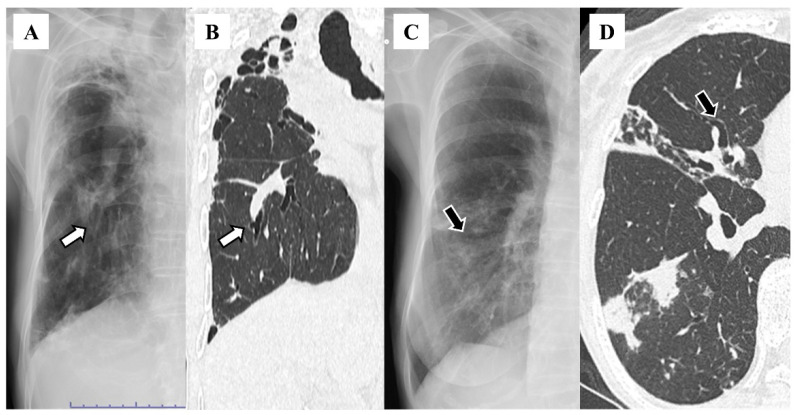
Tram-track appearance on chest X-ray (**A**) and high-resolution computed tomography (**B**). White arrows indicate the same lesions within the patient. Ring sign on chest X-ray (**C**) and high-resolution computed tomography (**D**). Black arrows indicate the same lesions within the patient.

**Figure 4:**
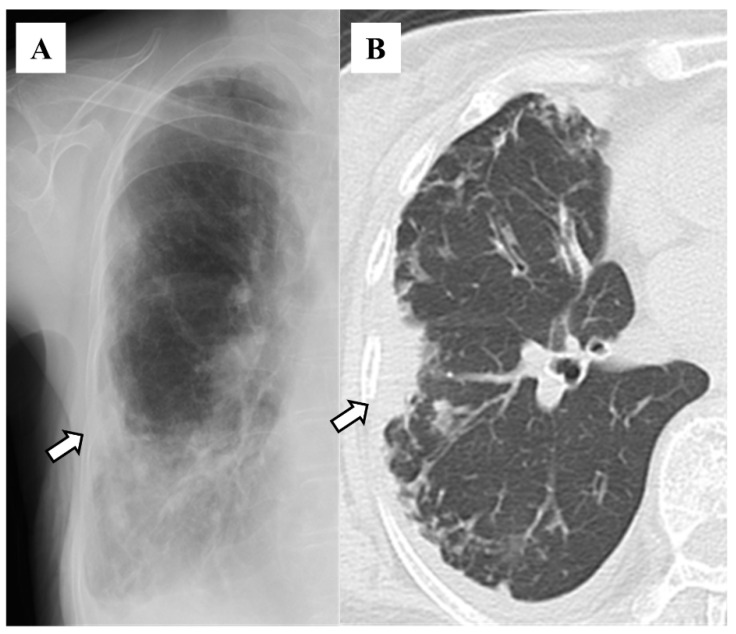
Pleural thickening on chest X-ray (**A**) and high-resolution computed tomography (**B**). White arrows indicate the same lesions within the patient.

The distributions of bronchiectasis features on HRCT included the right middle lobe (n = 83, 63.8%), left lingular segment (n = 75, 57.7%), right lower lobe (n = 58, 44.6%), right upper lobe (n = 56, 43.1%), left lower lobe (n = 45, 34.6%), and left upper segment (n = 22, 16.9%; Table 1). The features on chest X-rays included granular shadows (n = 115, 88.5%; Figure 1), the vague cardiac silhouette (n = 64, 48.5%; Figure 2), nodular shadows (n = 59, 45.4%; Figure 1), the tram-track appearance (n = 46, 35.4%; Figure 3), pleural thickening (n = 35, 26.9%; Figure 4), a vague diaphragm (n = 33, 25.4%; Figure 2), the ring sign (n = 32, 24.6%; Figure 3), lung volume loss (n = 15, 11.5%), the finger-in-glove sign (n = 12, 9.2%), atelectasis (n = 8, 6.2%), and the air–fluid level (n = 5, 3.8%; Table 2). The kappa value was 0.271 for granular shadows, 0.344 for vague cardiac silhouettes, 0.646 for nodular shadows, 0.256 for the tram-track appearance, 0.312 for pleural thickening, 0.514 for a vague diaphragm, 0.376 for the ring sign, 0.386 for lung volume loss, −0.015 for the finger-in-glove sign, 0.405 for atelectasis, and 0.359 for the air–fluid level. Comparing the sensitivity based on combinations of traditional features with that reported in the previous study (47%), 70 patients (53.8%) presented at least one of the following features: tram-track appearance, ring sign, air–fluid level, and finger-in-glove sign.

## 4. Discussion

This study demonstrated that X-ray features such as granular shadows and the vague cardiac silhouette were relatively common, whereas features that have traditionally been considered characteristic of bronchiectasis on chest X-rays, such as the tram-track appearance, ring sign, air–fluid level, and finger-in-glove sign, were less common. Currie et al. evaluated six features on chest X-rays, including “liner markings” (tram-track appearance in this study) and “bronchial wall thickening” (also ring sign), and they reported that two radiologists unanimously identified these features in 9 of 19 patients (47%) [13]. In their study, the frequencies of liner markings and bronchial wall thickening were 11% and 14%, respectively, in line with our findings that features that have traditionally been considered characteristic of bronchiectasis on chest X-rays are not commonly detected on chest X-rays.

Several reasons can be postulated to explain the low sensitivity of chest X-ray features for diagnosing bronchiectasis. First, chest X-rays generally have a lower resolution than CT, and a certain degree of bronchial wall thickening is required for its detection by X-rays. In addition, chest X-rays depict information from the chest to the back in a single image. Thus, other anatomical structures may overlap with the bronchial wall. Therefore, when multiple bronchiectatic lesions exist in the same cross-section, it is believed that chest X-rays can only evaluate them as opacity. The tram-track appearance is observed when the bronchi run perpendicular to the direction of the X-rays, whereas the ring sign is observed when the bronchi run parallel. Therefore, the ring sign is often observed around the pulmonary hilum, but it might not be evaluated because it overlaps with vascular shadows. In addition, the evaluation can be affected by radiographic conditions such as respiratory status and body size, which could also explain the low incidence of these features.

In this study, a vague cardiac silhouette, which has been hypothesized to be useful in bronchiectasis screening, was frequently observed among patients with bronchiectasis. It has been reported that in NTM lung disease [16,17], which is the main cause of bronchiectasis in Japan and the US, bronchiectatic features are more common in the middle lobe and lingular segment. The distributions of bronchiectatic findings on chest CT images in the current study were similar to these results. On chest X-rays, the middle lobe and lingular segment overlap with the cardiac silhouette. In fact, airway dilation in the right middle lobe and lingular segment was more common in patients with NTM lung diseases than in those with bronchiectasis caused by pulmonary diseases other than NTM [5]. Meanwhile, no specific distribution patterns of airway dilation have been revealed in patients with bronchiectasis associated with non-NTM. Therefore, a vague cardiac silhouette reflecting airway dilation in the right middle lobe and lingular segment might be commonly observed in patients with bronchiectasis caused by NTM lung diseases, but its usefulness in patients with other diseases remains unclear.

Conversely, in interstitial pneumonia, especially in idiopathic pulmonary fibrosis, lung opacity increases with interlobular septal thickening, which is typically distributed in both lower lobes [18]. This feature might also lead to the vague cardiac silhouette on chest X-rays, thereby reducing its specificity for diagnosing bronchiectasis. However, interstitial pneumonia is characterized by inflammation and fibrosis of the alveolar septa, which results in impaired lung expansion and a reduced lung volume [19]. The lung volume in patients with bronchiectasis is usually normal and rarely low [20,21]. In fact, the incidence of lung volume loss in this study was only 11.5%. Therefore, lung volume evaluation on chest X-rays could be useful for distinguishing between bronchiectasis and interstitial pneumonia among patients with a vague cardiac silhouette. For example, if a vague cardiac silhouette and preserved lung volume are simultaneously observed, it might be possible to proactively suspect bronchiectasis. Granular shadows are also frequently found among patients with bronchiectasis. Although granular shadows themselves are generally nonspecific features, they might have a higher positive predictive value in combination with a vague cardiac silhouette and preserved lung volume.

The strength of this study is that it evaluated chest X-ray findings in a large number of patients with bronchiectasis. However, some limitations must be discussed. First, this was a retrospective study, and it was limited to patients in whom bronchiectasis was identified on HRCT and in whom bronchoscopy was performed. This might have limited patients to those with a certain level of severity warranting active investigation into the cause of bronchiectasis (i.e., *Mycobacterium* infection), whereas FEV1% predicted mostly remained within the normal range. Therefore, the results of the current study might not be applicable to patients with early or mild bronchiectasis. Second, the average kappa for each feature, which indicates the rate of agreement in the interpretation of X-rays, was relatively low at around 0.35; thus, evaluation bias must also be considered. In general, the diagnostic agreement rate for chest X-rays has been reported to be low in evaluations of various respiratory diseases [22]. Third, the vague cardiac silhouette, which is believed to be attributable to lesions in the middle lobe and lingular segment, is likely to be a feature limited to areas in which NTM lung disease is common, such as Japan and the US. Therefore, the usefulness of this feature might vary in areas with a different prevalence of NTM lung disease. Fourth, this study only included patients diagnosed with bronchiectasis and investigated the sensitivity of radiological features on chest X-rays. Because no control group was included, the specificity and positive and negative predictive values of these radiological features remain unknown. However, it would be challenging to identify a disease that could serve as a counterpart to bronchiectasis. Finally, this study was conducted at a single university hospital, which likely accounted for the relatively severe cases that were more likely to be suitable for bronchoscopy. Moreover, patients’ ages in this study were relatively high, and caution should be exercised when generalizing the current results to other age groups.

In conclusion, features on chest X-rays that have traditionally been characteristic of bronchiectasis, such as the tram-track appearance and ring sign, were rarely observed, whereas a vague cardiac silhouette was frequently found. The vague cardiac silhouette is believed to be associated with lesions in the middle lobe or lingular segment. In areas with a high prevalence of NTM disease, these features might be useful for bronchiectasis screening and excluding interstitial pneumonia with lung volume loss.

## Figures and Tables

**Table 1:** The distributions of bronchiectasis features on high-resolution computed tomography.

Lobe	Number (%)
Right upper lobe	56 (43.1)
Right middle lobe	83 (63.8)
Right lower lobe	58 (44.6)
Left upper segment	22 (16.9)
Left lingular segment	75 (57.7)
Left lower lobe	45 (34.6)

Values are presented as number (%).

**Table 2:** The features on chest X-ray among patients with bronchiectasis.

Features	Number (%)
Granular shadows	115 (88.5)
Vague cardiac silhouette	63 (48.5)
Nodular shadows	59 (45.4)
Tram-track appearance	46 (35.4)
Pleural thickening	35 (26.9)
Vague diaphragm	33 (25.4)
Ring sign	32 (24.6)
Lung volume loss	15 (11.5)
Finger-in-glove sign	12 (9.2)
Air–fluid level	5 (3.8)
Atelectasis	8 (6.2)

Values are presented as number (%).

## Data Availability

The data are available from the corresponding author upon reasonable request.
